# Cholinergic imaging in dementia spectrum disorders

**DOI:** 10.1007/s00259-016-3349-x

**Published:** 2016-03-16

**Authors:** Roman Roy, Flavia Niccolini, Gennaro Pagano, Marios Politis

**Affiliations:** Neurodegeneration Imaging Group, Department of Basic & Clinical Neuroscience, Institute of Psychiatry, Psychology and Neuroscience, King’s College London, London, UK

**Keywords:** Cholinergic system, PET, SPECT, Dementia

## Abstract

The multifaceted nature of the pathology of dementia spectrum disorders has complicated their management and the development of effective treatments. This is despite the fact that they are far from uncommon, with Alzheimer’s disease (AD) alone affecting 35 million people worldwide. The cholinergic system has been found to be crucially involved in cognitive function, with cholinergic dysfunction playing a pivotal role in the pathophysiology of dementia. The use of molecular imaging such as SPECT and PET for tagging targets within the cholinergic system has shown promise for elucidating key aspects of underlying pathology in dementia spectrum disorders, including AD or parkinsonian dementias. SPECT and PET studies using selective radioligands for cholinergic markers, such as [^11^C]MP4A and [^11^C]PMP PET for acetylcholinesterase (AChE), [^123^I]5IA SPECT for the α_4_β_2_ nicotinic acetylcholine receptor and [^123^I]IBVM SPECT for the vesicular acetylcholine transporter, have been developed in an attempt to clarify those aspects of the diseases that remain unclear. This has led to a variety of findings, such as cortical AChE being significantly reduced in Parkinson’s disease (PD), PD with dementia (PDD) and AD, as well as correlating with certain aspects of cognitive function such as attention and working memory. Thalamic AChE is significantly reduced in progressive supranuclear palsy (PSP) and multiple system atrophy, whilst it is not affected in PD. Some of these findings have brought about suggestions for the improvement of clinical practice, such as the use of a thalamic/cortical AChE ratio to differentiate between PD and PSP, two diseases that could overlap in terms of initial clinical presentation. Here, we review the findings from molecular imaging studies that have investigated the role of the cholinergic system in dementia spectrum disorders.

## Introduction

The population of the developed world is ageing and this has led to an increasing prevalence of dementia. It has recently been estimated that 47.5 million people worldwide suffer from dementia [1]. Dementia is a challenge to society, both in terms of economic cost and social burden, as most patients require long-term care in their home or nursing home [2]. There is clearly a real incentive to find more effective methods of diagnosing and treating this set of diseases, which are not yet as fully understood as many of their other neurodegenerative diseases.

The cholinergic system plays a key role in functional and structural remodelling of cortical circuits underlying cognitive processing [3]. The three major cholinergic projection systems of the central nervous system include (1) the nucleus basalis of Meynert, which supplies cholinergic projections throughout the cerebral cortex and hippocampus [4], (2) the pedunculopontine nucleus pars compacta, which projects to the forebrain as well as various subcortical structures such as the thalamus [5], and (3) cholinergic neurons intrinsic to the striatum [6]. Post-mortem studies have found that muscarinic acetylcholine receptors (mAChR) are highly expressed in the caudate nucleus and nucleus accumbens in normal monkey and human brain [7–9]. In the somatosensory, primary motor and temporal cortices, mAChR concentrations are about 60 % of the levels in the caudate whilst the cerebellar cortex expresses the lowest levels [8]. Nicotinic acetylcholine receptors (nAChR) are abundantly expressed in the entorhinal, temporal and primary motor cortices, and the hippocampus and thalamus of post-mortem normal human brain tissue [10–12].

**Fig. 1:**
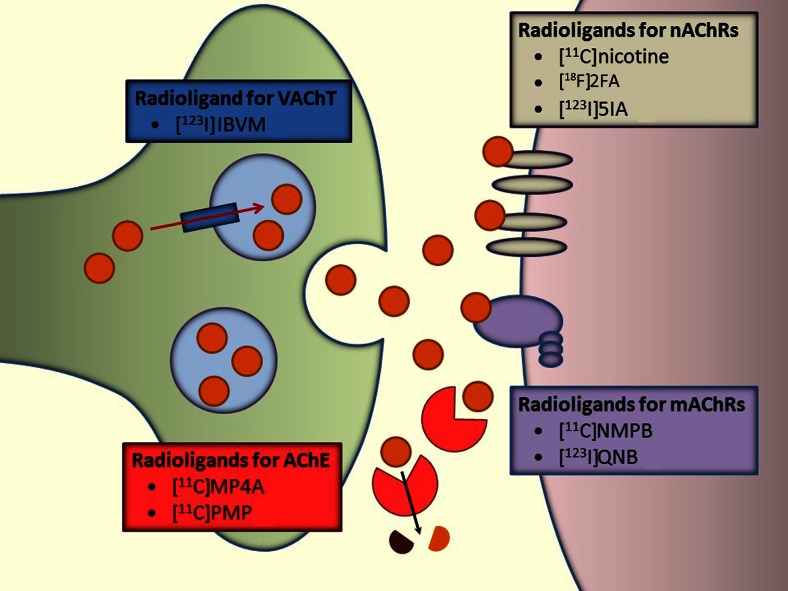
Schematic illustration of PET and SPECT techniques assessing presynaptic and postsynaptic cholinergic molecular targets with direct relevance to dementia. Acetylcholine (*ACh*) is degraded to choline and acetate by acetylcholinesterase (*AChE*), thereby terminating neurotransmission. The reuptake of choline into the presynaptic neuron occurs via a choline transporter. Choline is recycled within the presynaptic neuron to form ACh and stored in vesicles through a presynaptic vesicular ACh transporter (*VAChT*). Two different types of ACh receptors (*AChR*) are expressed on the postsynaptic neurons: nicotinic receptors (*nAChR*) and muscarinic receptors (*mAChR*)

Dysfunction of the ascending cholinergic systems from the basal forebrain and brainstem and the associated loss of cholinergic neurotransmission in the cerebral cortex has been suggested as an underlying substrate of cognitive decline (cholinergic hypothesis of dementia), supporting the use of acetylcholinesterase (AChE) inhibitors in dementia [13]. Many post-mortem studies investigating pathophysiological mechanisms of dementia have focused on alterations in functional components of the cholinergic system, such as AChE, the vesicular acetylcholine transporter (VAChT), nAChR, and mAChR [14, 15]. Molecular imaging techniques such as SPECT and PET with selective radioligands for targets within the cholinergic system have led to significant advances in the understanding of the neurobiology and pathophysiology of dementia (Fig. 1).

This review discusses the use of cholinergic radioligands in molecular imaging (Tables 1 and 2), with the aim of improving the understanding, and thereby the diagnosis, monitoring and treatment, of dementia spectrum disorders.

**Table 1:** Cholinergic PET and SPECT studies using presynaptic cholinergic markers in dementia spectrum disorders

Radiotracer	Target	Reference	Imaging technique	Subjects	Main findings
[^123^I]IBVM	VAChT	[16]	SPECT	22 AD, 9 PD, 6 PDD, 36 HC	Significant reduction in VAChT levels in entire cortex in PDD; only reduced in parietal and occipital cortex in PD. In mildly demented AD patients with age of onset <65 years, severe reduction in cortical and hippocampal binding. In AD patients with age of onset >65 years, VAChT reductions only in temporal cortex and hippocampus.
[^11^C]PMP	AChE	[17]	PET	14 AD, 26 HC	Significant reduction in cortical AChE activity in AD patients. No significant changes in AChE activity in caudate, putamen, thalamus, pons or cerebellum.
[^11^C]MP4A	AChE	[18]	PET	16 PD, 12 PSP, 13 HC	Significant reduction in cortical AChE activity in PD (−17 %); no significant changes in thalamic [^11^C]MP4A uptake in PD. Significant reduction in thalamic AChE activity in PSP (−38 %) but no significant change in cortical binding. Possible use of thalamic to cortical [^11^C]MP4A binding ratio for the differential diagnosis of PD and PSP.
[^11^C]PMP	AChE	[19]	PET	12 AD, 11 PD, 14 PDD, 10 HC	Highest reduction in cortical AChE activity in PDD (−20 %), then PD (−12.9 %), and least in AD (−9.1 %). Selective involvement of lateral temporal cortex in AD (−15 %).
[^11^C]PMP	AChE	[20]	PET	15 AD, 12 HC	Significant reduction in cortical AChE activity in AD patients. Positive correlation between cortical AChE activity and attention and working memory; no correlation with short-term or long-term memory.
[^11^C]MP4A	AChE	[21]	PET	17 PD, 10 PDD, 31 HC	Larger reduction in cortical AChE in PDD (−29.7 %) than in PD (−10.7 %). Close relationship between striatal [^18^F]FDOPA binding and [^11^C]MP4A binding in frontal and temporoparietal cortex in PDD.
[^11^C]MP4A	AChE	[22]	PET	11 AD ApoE4+, 8 AD ApoE4−	Larger reduction in AChE activity in ApoE4− than ApoE4+ patients.
[^11^C]PMP	AChE	[23]	PET	13 PD, 11 PDD, 14 HC	Significant reduction in cortical AChE activity in PDD (−20.9 %) and PD (−12.7 %). Significant correlation between cortical AChE and WAIS-III Digit Span test, Trail Making test and Stroop Color Word test scores in PDD. No significant correlation between motor symptom severity and cortical binding.
[^11^C]MP4A	AChE	[24]	PET	18 PD, 10 PDD, 11 DLB, 26 HC	Significant reduction in cortical AChE activity in PDD and DLB (−27 %); no significant difference between PDD and DLB groups. Significant reduction in cortical binding in PD, especially in medial occipital cortex (−12 %); significant difference between PDD and DLB, and PD groups.
[^11^C]PMP	AChE	[25]	PET	12 PD, 13 MSA, 4 PSP, 22 HC	Significant reduction in AChE activity in most cortical regions in PD (−15.3 %) and MSA (−14.6 %); no significant differences between PD and MSA groups. No significant changes in cortical binding in PSP. Preferential denervation of subcortical structures (striatum, cerebellum, thalamus, midbrain and pons) in MSA and PSP. Significant reduction in striatal, cerebellar, thalamic binding in PD, but significantly smaller reduction than in MSA and PSP.
[^11^C]MP4A	AChE	[26]	PET	9 PD, 8 PDD, 6 DLB, 3 HC	Severe reduction in neocortical AChE activity in PDD and DLB, with the reduction increasing from frontal (least) to occipital cortex (most) in both groups. No significant difference between PDD and DLB groups. Mild cortical AChE deficit in PD.
[^11^C]MP4A	AChE	[27]	PET	7 CBD, 12 PSP, 8 FTD, 16 HC	Voxel-based analysis showed significant decreases in AChE activity in CBD (paracentral region, frontal, parietal and occipital cortices) and PSP (paracentral region and thalamus). No significant differences in [^11^C]MP4A binding in FTD. Volume of interest analysis showed mean cortical AChE activity reduced by 17.5 % in CBD, 9.4 % in PSP and 4.4 % in FTD; thalamic AChE activity reduced by 24.0 % in PSP but not in CBD and FTD.
[^11^C]PMP	AChE	[28]	PET	13 AD, 11 PD, 6 PDD, 6 DLB, 14 HC	Largest reduction in thalamic AChE activity in PDD (−19.8 %), then in DLB (−17.4 %), least in PD (−12.8 %). Spared thalamic AChE activity in AD.
[^123^I]IBVM	VAChT	[29]	SPECT	10 PSP, 12 HC	Significant reduction in anterior cingulate cortical, innominatocortical, thalamic and pontothalamic VAChT levels Thalamic and pedunculopontine binding inversely correlated with disease duration.

*AChE* acetylcholinesterase, *AD* Alzheimer’s disease, *CBD* corticobasal degeneration, *DLB* dementia with Lewy bodies, *FTD* frontotemporal dementia, *HC* healthy control, *MSA* multiple system atrophy, *PD* Parkinson’s disease, *PDD* Parkinson’s disease dementia, *PSP* progressive supranuclear palsy, *VAChT* vesicular acetylcholine transporter

**Table 2:** Cholinergic PET and SPECT studies using postsynaptic cholinergic markers in dementia spectrum disorders

Radiotracer	Target	Reference	Imaging technique	Subjects	Main findings
[^11^C]NMPB	mAChR	[30]	PET	7 PSP, 12 PD, 8 HC	Significant increase in mAChR in frontal cortex in PD (22 %). No significant change in any cortical region in PSP.
[^123^I]QNB	mAChR	[31]	SPECT	25 PDD, 14 DLB, 24 HC	Significant increase in mAChR in right occipital lobe in DLB, occipital lobes bilaterally in PDD; no significant difference between DLB and PDD. Significant reduction in mAChR expression in frontal and temporal lobes in PDD, nonsignificant in DLB.
[^11^C]Nicotine	nAChR	[32]	PET	27 AD, 36 HC	Significant correlation between cortical nAChR expression and Digit Symbol test score, as well as Trail Making Test A score.
[^123^I]5IA	α4β2 nAChR	[33]	SPECT	10 PD, 15 HC	Significant widespread reduction in cortical and subcortical α2β4 nAChR levels in PD (−10 %); highest in thalamus (−15 %), parietal cortex (−9 %) and temporal cortex (−8 %); lowest in frontal cortex (−5 %) and occipital cortex (−3 %). No significant change in K1 (index of radiotracer delivery).
[^123^I]5IA	α4β2 nAChR	[34]	SPECT	16 AD, 16 HC	Significant reductions in α2β4 nAChR expression in frontal, striatal, right medial temporal and pontine regions in AD.
[^123^I]5IA	α4β2 nAChR	[35]	SPECT	10 PD, 10 HC	Significant reductions in brainstem and frontal cortex α2β4 nAChR levels in PD (−20 – 25 %). Significant negative correlation between high daily dose of dopamine agonist and tracer binding in cerebellum and temporal, parietal and occipital cortices.
[^18^F]2FA	α4β2 nAChR	[36]	PET	15 AD, 14 HC	No significant change in α2β4 nAChR in early AD compared to HC; suggestive of nAChR preservation in early stages of AD.
[^123^I]5IA	α4β2 nAChR	[37]	SPECT	15 DLB, 16 HC	Significant reduction in α2β4 nAChR levels in frontal, temporal, cingulate cortices and striatum. Significant increase in occipital cortex expression, correlated with visual hallucinations.
[^18^F]2FA	α4β2 nAChR	[38]	PET	17 AD, 6 MCI, 10 HC	Significant reduction in α2β4 nAChR expression in thalamus, striatum, cerebellum and cortex of both AD and MCI patients. Significant negative correlation between [^18^F]2FA binding and level of cognitive impairment.
[^18^F]2FA	α4β2 nAChR	[39]	PET	13 PD, 6 HC	Significant reduction in α2β4 nAChR in striatum (−10 %) and substantia nigra (−14.9 %) in PD.
[^18^F]2FA	α4β2 nAChR	[40]	PET	22 PD, 9 HC	Significant reduction in α2β4 nAChR levels, most pronounced in the midbrain, pons, anterior cingulate and frontoparietal cortices, and cerebellum. Significant correlation between [^18^F]2FA binding in the anterior cingulate and occipital cortices and depression. Significant correlation between [^18^F]2FA binding in the midbrain, pons and cerebellum and cognitive impairment.
[^123^I]5IA	α4β2 nAChR	[41]	SPECT	12 AD, 10 MCI, 10 HC	No significant difference in α2β4 nAChR levels in any brain region investigated (frontal, parietal, anterior cingulate, temporal and occipital cortices, thalamus, striatum, cerebellum).
[^123^I]5IA	α4β2 nAChR	[42]	SPECT	10 MCI, 10 HC	Significant reduction in tracer binding in medial temporal cortex. Significant correlation between [^123^I]5IA uptake in left temporoparietal cortex, bilateral temporolimbic areas and right parahippocampal gyrus and level of cognitive impairment.
[^18^F]2FA	α4β2 nAChR	[43]	PET	20 AD, 25 HC	Significant reduction in α2β4 nAChR availability in thalamus, caudate, and prefrontal cortex, and medial and lateral temporal cortices. Significant positive correlation between [^18^F]2FA uptake in medial frontal cortex and nucleus basalis of Meynert and Frontal Assessment Battery test scores in AD patients. Significant negative correlation between amyloid-β load and α2β4 nAChR expression in frontal cortex.

*AD* Alzheimer’s disease, *DLB* dementia with Lewy bodies, *HC* healthy control, *mAChR* muscarinic acetylcholine receptor, *MCI* mild cognitive impairment, *nAChR* nicotinic acetylcholine receptor, *PD* Parkinson’s disease, *PDD* Parkinson’s disease dementia, *PSP* progressive supranuclear palsy

## Cholinergic system in Alzheimer’s disease

AD is the leading cause of dementia in the western world, accounting for more than 60 % of cases [44, 45]. AD is characterized pathologically by accumulation of amyloid β peptide (Aβ) in extracellular plaques, intracellular deposits of tau protein, and neuronal loss [46–48]. Evidence for the involvement of the cholinergic system in the pathogenesis of AD was provided as early as the mid-1970s in post-mortem studies showing loss of choline acetyltransferase (ChAT) and AChE in the cortex, hippocampus and amygdala of AD brain samples [14, 49–50]. Moreover, reduced ChAT activity has been found to be correlated with increased Aβ plaque load and with cognitive decline [50, 52]. Degeneration of the cholinergic system affects not only cortical regions but also the nucleus basalis of Meynert, where cholinergic neurons are severely decreased in post-mortem brain tissue of AD patients, highlighting the role of subcortical cholinergic dysfunction in the pathogenesis of AD [53].

### Presynaptic cholinergic dysfunction in Alzheimer’s disease

PET with *N*-[^11^C]methyl-piperidin-4-yl propionate ([^11^C]PMP), a selective substrate for AChE [54], has shown a reduction in AChE activity in AD patients [17, 19, 20]. The degree of AChE activity reduction ranged between 9 % and 33 %, depending on the severity of cognitive impairment in the cohort of AD patients examined. Kuhl et al. [17] found decreases in neocortical and hippocampal AChE activity of 25 – 33 % in patients with moderate–severe AD and a mean Mini Mental State Examination (MMSE) score of 14. Loss of cortical [^11^C]PMP uptake is associated with reductions in VAChT, as measured by 5-[^123^I]iodo-benzovesamicol ([^123^I]IBVM) SPECT, but does not correlate with decreases in 2-[^18^F]-fluoro-2-deoxy-d-glucose ([^18^F]FDG) PET, which is focally reduced in the posterior cingulate gyrus and parietal cortex [17]. The disagreement in cholinergic and metabolic imaging patterns of degeneration suggests a different mechanism underlying cholinergic and metabolic decline in AD pathophysiology. Patients with early AD (mean MMSE score 22) showed more modest reductions in cortical AChE activity (9 – 11 %) with greater decreases in the lateral temporal cortex (15 %) [19, 20]. Overall, in vivo AChE losses tend to be less pronounced than those seen in post-mortem studies, in which 55 % reductions in cholinergic markers have been observed [55].

The modest degree of cholinergic denervation despite the severity of cognitive decline raises questions about the specific role of the cholinergic system in episodic memory processes. In this regard, Bohnen et al. [20] found that decreases in cortical AChE activity were negatively correlated with performance in the WAIS-III digit span test, whereas they were not associated with California Verbal Learning Test (CVLT) scores. These results suggest that cholinergic dysfunction is linked to attention and working memory rather than episodic memory. Although cortical cholinergic denervation from the nucleus basalis of Meynert is a feature of AD, the pontine cholinergic projection system to the thalamus has been found to be spared in the disease [28].

PET with *N*-[^11^C]methyl-4-piperidyl acetate ([^11^C]MP4A), another selective AChE radioligand, has shown that AD patients with apolipoprotein E allele ɛ4 (ApoE4) have significantly less pronounced reductions in cortical AChE activity than those negative for the ApoE4 allele [22]. This suggests that the ApoE4 allele has a protective role against the widespread loss of AChE activity in AD, although the underlying mechanism remains unknown. [^11^C]MP4A has shown higher specificity for AChE relative to butyryl cholinesterase [56, 57], but it has a higher hydrolysis rate and thus radioligand uptake in regions of high AChE activity such as basal ganglia is strongly dependent on the rate of transport into the brain [54]. In contrast, [^11^C]PMP exhibits a rate of hydrolysis three to four times slower than that of [^11^C]MP4A, allowing more precise estimates of the AChE activity in regions of moderate to high AChE concentration [58].

SPECT with [^123^I]IBVM, a selective ligand for presynaptic VAChT, has been used to assess presynaptic cholinergic terminal density [59]. Interestingly, in mildly demented AD patients, the [^123^I]IBVM binding pattern differs according to age at onset [16]. In patients with early-onset AD (age at onset <65 years) loss of cholinergic terminals were observed in the neocortex and hippocampus, whereas in patients with late-onset AD significant VAChT reductions were limited to the temporal cortex and hippocampus. These findings are consistent with those of post-mortem studies, which have demonstrated more widespread cholinergic degeneration in early-onset AD [60]. A new PET radioligand with good sensitivity and specificity for VAChT, (−)5-^18^F-fluoroethoxybenzovesamicol ([^18^F]FEOBV), has recently been developed and tested in humans [61]. Both reference tissue modelling and late static scanning approaches correlated well with the full kinetic modelling with arterial sampling and plasma metabolite analysis [61]. In comparison with [^123^I]IBVM SPECT, [^18^F]FEOBV PET showed low binding in the lateral cerebellar cortex and high binding in the mesopontine junction and medulla, providing a robust index of VAChT binding [61]. [^18^F]FEOBV PET is a significant advance over currently available presynaptic cholinergic imaging agents and may be a valuable tool to assess dysregulation of the cholinergic system in AD and parkinsonian patients.

### Postsynaptic cholinergic dysfunction in Alzheimer’s disease

Previous PET studies using [^11^C]nicotine have shown significant reductions in nAChR binding in the frontal cortex, temporal cortex and hippocampus of patients with moderate AD [62, 63]. Similar to AChE activity, decreases in cortical nAChR expression as measured by [^11^C]nicotine are associated with attention deficit but not episodic memory impairment [32]. These [^11^C]nicotine studies, however, were hindered by high levels of nonspecific binding, rapid metabolism, and washout from the brain, as well as a strong dependence on cerebral blood flow [64, 65]. More recently, new PET and SPECT radioligands have been developed to target α_4_β_2_ nAChR. Post-mortem autoradiography studies have shown that α_4_β_2_ nAChR is the most affected receptor subtype in AD, with reductions of up to 50 % in the neocortex, entorhinal cortex and hippocampus [12, 66, 67]. [^123^I]5-iodo-3-[2(*S*)-2-azetidinylmethoxy] pyridine ([^123^I]5IA) SPECT has shown significant reductions in α_4_β_2_ nAChR in the frontal cortex, striatum, right medial temporal lobe and pons of AD patients [34]. [^123^I]5IA binding is also significantly reduced in the medial temporal cortex of patients with amnestic mild cognitive impairment (MCI) and α_4_β_2_ nAChR decreases correlated with cognitive decline [42]. However, other studies have yielded contrasting results, finding no changes in α_4_β_2_ nAChR binding in cortical and thalamic regions of patients with early AD or MCI compared to healthy controls [36, 41]. This discrepancy may be due to the different severities of cognitive impairment of the subjects studied and different methodologies in assessing α_4_β_2_ nAChR expression.

PET with 2-[^18^F]F-A-85380 ([^18^F]2FA), a radioligand selective for α_4_β_2_ nAChR, has shown significant reductions in nAChR availability in the hippocampus, and frontal, temporal and parietal cortices of AD patients, which furthermore correlated with the level of cognitive impairment [38]. Another [^18^F]2FA study showed significantly reduced α_4_β_2_ nAChR expression in the medial frontal cortex and nucleus basalis of Meynert in AD patients, which were correlated with Frontal Assessment Battery scores, suggesting that in vivo α_4_β_2_ nAChR plays role in those specific functions that may be different from episodic memory [43]. Furthermore, a negative correlation between α_4_β_2_ nAChR availability and Aβ load (measured by [^11^C]Pittsburgh compound B) was found in the same brain regions, suggesting that Aβ deposition may induce degeneration of cholinergic neurons [43]. However, [^18^F]2FA exhibits slow brain distribution kinetics and relatively low binding potentials (≤0.6 – 0.8) in extrathalamic regions including the cortex which are of high importance in studying neurodegenerative diseases [68]. In recent years, major effort has focused on the development of new α_4_β_2_ nAChR PET radioligands with faster regional brain kinetics than [^18^F]2FA that will enable further imaging studies in dementia spectrum disorders [69].

### Cholinergic imaging in assessing Alzheimer’s disease treatment

Cholinergic PET imaging techniques have also been employed to assess the efficacy of medications. PET with [^11^C]PMP has shown 19 – 39 % inhibition of cortical AChE activity following donepezil treatment in AD patients [70, 71] whereas 28 – 39 % AChE inhibition has been observed after donepezil and rivastigmine in the frontal temporal and parietal regions of AD patients using [^11^C]MP4A PET [72, 73]. Kadir and colleagues [74] investigated the effect of galantamine, a cholinesterase inhibitor, on AChE activity and nicotine binding to nAChR in AD patients. They found significant reductions in cortical [^11^C]PMP following galantamine treatment at 3 weeks, and 3 and 12 months compared with baseline [74]. These findings suggest that galantamine treatment is able to increase ACh concentrations in the synaptic cleft resulting in increased cholinergic neurotransmission. No significant changes in [^11^C]nicotine binding were found following galantamine treatment [74]. Galantamine acts on nAChR as an allosterically potentiating ligand [75], thereby sensitizing the receptors, which in turn causes nAChR upregulation to maintain a baseline level of signalling.

## Cholinergic system in parkinsonian dementias

Parkinson’s disease (PD) is a chronic neurodegenerative disorder characterized pathologically by degeneration of dopaminergic neurons in the substantia nigra pars compacta and formation of α-synuclein proteinaceous intraneuronal inclusions referred to as Lewy bodies and Lewy neurites [76–78]. In addition to classical motor symptoms, non-motor features such as cognitive decline, are a very important aspect of the disease, because they add significantly to the burden on patients and caregivers [79, 80]. PD patients carry a sixfold increased risk for dementia compared to the general population, with approximately 80 % of patients developing PD dementia (PDD) over the course of the disease [81, 82]. MCI in PD also appears to be common, occurring in 20 % to 50 % of PD patients, even at the time of PD diagnosis and prior to initiation of dopaminergic therapy [83, 84]. Dementia with Lewy bodies (DLB) is characterized by fluctuating cognitive and attentional deficits, recurrent visual hallucinations and parkinsonism, and is the second most common cause of degenerative dementia, accounting for 15 – 25 % of cases [85]. Evidence for the involvement of the cholinergic system in the development of PDD and DLB has been provided by post-mortem studies, which have shown decreased AChE activity and nAChR density in cortical and subcortical brain tissue of PDD and DLB patients [86–92].

### Presynaptic cholinergic dysfunction in parkinsonian dementias

PET studies using [^11^C]MP4A and [^11^C]PMP have demonstrated mild to moderate decreases in cortical AChE activity in PD patients without dementia, and severe decreases in PDD and DLB patients [21, 23, 26]. Using SPECT with [^123^I]IBVM, Kuhl et al. [16] demonstrated that whilst in PD without dementia, VAChT levels are reduced only in the parietal and occipital cortices, in PDD, major losses are seen throughout the entire cortex, suggesting a greater and more widespread presynaptic cholinergic dysfunction in PDD patients. Loss of AChE activity occurs in early stages of the disease, with de novo PD patients showing significant (12 %) AChE losses in the medial occipital cortex [24]. Differences in AChE activity between early and advanced PD are nonsignificant, however, suggesting that cholinergic dysfunction occurs early, but does not progress with the disease [24]. Moreover, loss of cortical AChE activity as measured by [^11^C]PMP is correlated with worse performance in working memory and attention tests but not with the severity of motor symptoms [23]. A relationship between striatal [^18^F]FDOPA uptake and cortical [^11^C]MPA reduction has been found in PDD patients, suggesting that cognitive decline in PD occurs when the disease spreads from nigral neurons to the cortex, leading to a cholinergic dysfunction in this region [21].

Cholinergic dysfunction is higher in PDD patients than in AD patients with a similar degree of cognitive impairment (MMSE score 22.8 and 22.2, respectively) suggesting that the mechanisms underlying cognitive decline in PD and AD have different pathogeneses [19]. In contrast to the cholinergic system in AD, which was spared (0.7 % reduction), thalamic AChE activity was found to be decreased by 19.8 % in PDD patients [28].

Cortical AChE activity differs between patients with PD and those with progressive supranuclear palsy (PSP) [18, 25]. PD patients show significant decreases in cortical [^11^C]MP4A uptake, whereas PSP patients show significant reductions only in the thalamus [18]. Therefore, a thalamic/cortical [^11^C]MP4A binding ratio may be useful in distinguishing between PD and PSP. A SPECT study using an [^123^I]IBVM ligand found that VAChT levels are significantly lower in the thalamus and anterior cingulate cortex of PSP patients compared to healthy controls, and that thalamic cholinergic dysfunction is inversely correlated with disease duration [29]. This is in line with the findings of similar PET studies reported previously, that show a preferential cholinergic denervation in the thalamus of PSP patients [83]. VAChT levels, however, are unaffected in the striatum of PSP patients [29]. This differs from PD pathology, in which cholinergic neurons in the striatum are affected, suggesting a possible role of cholinergic imaging markers in aiding the differential diagnosis of these two clinically similar diseases [29].

 In other atypical parkinsonisms such as multiple system atrophy, cortical AChE activity decreases have been found to be similar to those seen in PD patients (−14.6 % and −15.3 %, respectively) [25]. Hirano et al. [27] investigated differences in AChE activity in patients with PSP, corticobasal degeneration (CBD) and frontotemporal dementia (FTD) using both voxel-based and volume of interest analysis. Statistical parametric mapping analysis showed significant AChE decreases in the paracentral region, frontal, parietal and occipital cortices in CBD patients and in the paracentral region and thalamus in PSP patients [27]. FTD patients showed no significant differences in AChE activity compared to the control group. Volume of interest analysis showed significant decreases in thalamic AChE activity only in PSP patients. Thus, cholinergic dysfunction occurs in CBD and PSP, although involving different brain regions, but it was not observed in FTD patients, which may explain why AChE inhibitors are ineffective for this condition [93].

### Postsynaptic cholinergic dysfunction in parkinsonian dementias

Post-mortem studies have shown that cortical mAChR density is altered in PDD and DLB samples [94, 95]. An in vivo PET study with *N*-[^11^C]methyl-4-piperidyl benzilate ([^11^C]NMPB), a marker for mAChR, demonstrated increased mAChR levels in the frontal cortex of PD patients, probably due to denervation hypersensitivity caused by loss of the ascending cholinergic system in frontal areas [30]. Contrastingly, in PSP patients, cortical and thalamic [^11^C]NMPB binding was preserved [30]. A SPECT study using [^123^I]iodo-quinuclidinyl benzilate ([^123^I]QNB), another marker for mAChR, showed significant increases in mAChR in the occipital lobe in both PDD and DLB, which may be the substrate of visual disturbances in these diseases [31]. Furthermore, there was significantly lower [^123^I]QNB binding in the frontal and temporal lobes of PDD patients than in DLB patients. [^11^C]NMPB and [^123^I]QNB are high-affinity mAChR antagonists with similar chemical structures and regional brain distributions [96–98]. Both radioligands penetrate the blood–brain barrier efficiently but nonspecifically in relation to mAChR subtype [99].

[^123^I]5IA SPECT studies have shown 10 – 25 % reductions in nAChR levels in cortical and subcortical regions of PD patients [33, 95], with the largest decreases observed in the thalamus, and parietal and temporal cortices [33]. A [^18^F]2FA PET study showed significant reductions in α_4_β_2_ nAChR availability in various regions in PD patients, including the frontoparietal and anterior cingulate cortices, midbrain, pons and cerebellum, with the highest reduction in the left parietal cortex [40]. Furthermore, α_4_β_2_ nAChR loss correlated with depression and cognitive decline as measured by the MMSE, the DemTect Scale, the Clock Drawing test, delayed recall of figures of the CERAD (Consortium to Establish a Registry for Alzheimer’s Disease) battery, and the Trail Making test [40]. In nondemented PD patients, cortical and subcortical [^123^I]5IA decreases correlated with worse performance In the Boston Naming test and Word List Intrusions test, two specific tests for detection of word-finding difficulties, learning capacity and memory for language information [98]. Decreases in [^18^F]2FA uptake were also found in the striatum (−10 %) and substantia nigra (−14.9 %) of PD patients, but did not correlate with dopaminergic function as measured by [^18^F]F-DOPA, nor with clinical severity [39].

 [^123^I]5IA SPECT has shown significant reductions in α_4_β_2_ nAChR binding in the frontal, temporal and cingulate cortex, as well as in the striatum of DLB patients [37]. In the occipital cortex, however, α_4_β_2_ nAChR levels were increased and correlated with visual hallucinations experienced by DLB patients, suggesting cholinergic dysfunction in the occipital lobe as a substrate of visual hallucinations. Because α_4_β_2_ nAChRs are located in some regions presynaptically on both cholinergic and noncholinergic terminals [101], some difficulties may arise in the interpretation of nAChR imaging studies. In PD, significantly decreased α_4_β_2_ nAChR levels may be a result of nAChR loss on degenerated presynaptic nigrostriatal dopaminergic neurons. A post-mortem study has shown that loss of striatal nAChR binding closely parallels the loss of nigrostriatal dopaminergic markers in PD brain tissue [102]. Postsynaptic nAChR receptors may show a compensatory increase, no change, or a decrease due to degeneration of noncholinergic systems (e.g. noradrenergic, serotoninergic, glutamatergic) to which the nAChRs are coupled [37, 102]. Further studies combining cholinergic and noncholinergic radioligands may aid in elucidating the nAChR alteration patterns in parkinsonian disorders.

## Conclusion

Cholinergic dysfunction has a pivotal role in the pathophysiology of cognitive decline. Given the wide variety of available markers for the cholinergic system, molecular imaging techniques provide a valuable tool to investigate pathophysiological mechanisms, and monitor progression and response to treatments in AD and parkinsonian dementia. The largest drawback of this technology, its cost, will hopefully be improved by its widening use as it becomes increasingly clear that PET and SPECT imaging holds much potential for gleaning important information about the pathology of dementia. Much remains to be learned about cholinergic dysfunction in AD, PD and related disorders. PET studies combining presynaptic and postsynaptic radioligands may be useful in unravelling alterations of cholinergic neurotransmission. Moreover, it has been suggested that the α7 nAChR subtype may have a neuroprotective role by modulating the neurotrophic system crucial for the maintenance of cholinergic neuron integrity, and also by stimulating signal transduction pathways that support neuron survival [103]. In AD, α7 nAChR may modulate β-amyloid-induced pathology [104], and deletion of the α7 nAChR gene has shown to improve cognitive impairment in animal models of AD [105]. Further PET studies using radioligands specific to the α7 nAChR such as [^18^F]ASEM [106, 107] are needed to determine the relationship between α7 nAChR and AD pathology.
